# Only incandescent light significantly decreases feeding of *Anopheles funestus* s.s. (Diptera: Culicidae) mosquitoes under laboratory conditions

**DOI:** 10.1007/s00436-024-08370-3

**Published:** 2024-10-18

**Authors:** Layla van Zyl, Ashley M. Burke, Lizette L. Koekemoer, Bernard W. T. Coetzee

**Affiliations:** 1 Department of Zoology and Entomology, University of Pretoria, Pretoria, South Africa; 2 Wits Research Institute for Malaria, Faculty of Health Sciences, University of the Witwatersrand, Johannesburg, South Africa; 3 Centre for Emerging Zoonotic and Parasitic Diseases, National Institute for Communicable Diseases of the National Health Laboratory Service, Johannesburg, South Africa

**Keywords:** ALAN, LED light, Blood feeding, Malaria

## Abstract

Recent work has demonstrated that exposure to artificial light at night (ALAN) may alter mosquito feeding behavior and so must be considered a moderator of vector-borne disease transfer. *Anopheles funestus* mosquitoes are a primary malaria vector in sub-Saharan Africa, but no study to date has tested the impact of ALAN on their feeding behavior. Here we test if the exposure to commonly used household lights (compact fluorescent lights, light-emitting diodes, and incandescent lights) alters *Anopheles funestus* feeding. Mated, unfed female mosquitoes were exposed to a light treatment, at the onset of darkness, followed by a blood-feeding assay. The light treatments consisted of a 30-min light pulse of one of the three household lights, each in individual experimental containers, versus controls. All three household lights resulted in a reduction in the percentage of females taking a blood meal, but only mosquitoes exposed to incandescent light showed a statistically significant reduction in feeding of 19.6% relative to controls which showed a 42.8% feeding rate. Our results suggest that exposure to some household lights during the night may have an immediate inhibitory effect on *Anopheles funestus* feeding. By helping identify which light types lead to a suppression of feeding, the findings of this study could provide insight necessary to design household lights that can help minimize mosquito feeding on humans.

**Supplementary information:**

The online version contains supplementary material available at 10.1007/s00436-024-08370-3.

## Background

Vector-borne diseases remain a cause of public health concern. Malaria caused an estimated 249 million cases and ~ 608,000 deaths in 2022 (WHO 2023). Current vector control strategies include the use of insecticide-treated bed nets and indoor residual spraying (WHO 2015; Scates et al. 2020; Tangena et al. 2020). These strategies, however, are reducing in effectiveness due to insecticide resistance (Coetzee and Koekemoer 2013; Ranson and Lissenden 2016), highlighting the increasing importance of exploring alternative disease transfer suppression strategies, such as the use of artificial light or genome editing technologies (Sheppard et al. 2017; Quinn et al. 2021).

The use of artificial light at night (ALAN) is increasing globally (Sánchez de Miguel et al. 2021) and has recently been demonstrated to alter the feeding frequency across a range of mosquito species. ALAN not only affects organism physiology, but also increases the overlap in periods in which both humans and nocturnal mosquitoes are active, providing more transmission opportunities for the parasite and so, possibly, leading to an increase in malarial infections (Rund et al. 2016). Rund et al. (2020) showed that light pulses change the feeding frequency of day-biting *Aedes* mosquitoes, where exposure to ALAN leads to an increase in night-time feeding by the diurnal mosquito. Sheppard et al. (2017) demonstrated that pulses of artificial light reduce the night-time biting rate of nocturnal *Anopheles* (*An*.) *gambiae* sensu stricto (s.s.) mosquitoes. These observations suggest that ALAN increases the feeding frequency of diurnal mosquitoes while reducing that of nocturnal mosquitoes (Coetzee et al. 2022). Therefore, while ALAN may increase contact between humans and nocturnal vectors and so lead to increased malaria transmission opportunities, ALAN also has the potential to decrease transmission by reducing the feeding of certain mosquito species. How and by which mechanisms ALAN alters the behavior of mosquitoes, however, remains to be investigated further.

*Anopheles funestus* s.s. (called *An. funestus* going forward) has a wide distribution across the tropical regions of Africa (Gillies and De Meillon 1968). These mosquitoes show a strong preference for human hosts and indoor resting spaces and are highly efficient vectors of malaria (Gillies and De Meillon 1968; Odero et al. 2023). *Anopheles funestus* are nocturnal feeders, with feeding times commencing around 18h00 with relatively low activity, which steadily increases from 21h00 and reaches a plateau of maximal feeding from 02h00 (Gillies and De Meillon 1968; Rund et al. 2013; Sande et al. 2016). Nocturnal *An. funestus* was chosen as the focus of this study due to its increasing epidemiological importance as a major malaria vector in sub-Saharan Africa (Hunt et al. 2005; Odero et al. 2023; Msugupakulya et al. 2023), and that, as far as we are aware, the impact of ALAN on its feeding behavior has not been investigated.

Laboratory-based experiments on mosquito feeding behavior commonly make use of lights with constant emission spectra at intensities not often used by humans and are, therefore, not an accurate representation of typical light conditions at the household scale. The main light types used in households include incandescent, compact fluorescent lights (CFLs), and light-emitting diodes (LEDs) (Wilson et al. 2021). Of the common household lights, incandescent lights are the least energy-efficient and LEDs are the most energy-efficient (Wilson et al. 2021). With LEDs being the cheapest option, these lights have become popular and it is expected that their use in Africa will show the largest increase over time (Cohnstaedt et al. 2008; Wilson et al. 2021). As LEDs are becoming more widely used, it is important to understand the effect that these lights have on African vectors such as *An. funestus*, particularly in areas where vector-borne disease burden is already high (Wilson et al. 2021).

Here we test how artificial lights commonly used in households (namely LED, CFL, and incandescent lights) alter *An. funestus* feeding frequency under laboratory conditions. As *An. funestus* is a night feeder, we hypothesize that exposure to ALAN will lead to a reduction in the percentage of blood-fed females (Coetzee et al. 2022). Other mosquito genera have sensitivity peaks in the UV and blue/green spectra (Wilson et al. 2021). Therefore, we predict that of the three household lights used, LED lights which have peaks in the blue and green spectra will have the greatest effect on reducing *An. funestus* feeding.

## Methods

### Assessing experimental conditions

To test the effect of ALAN on *An. funestus* feeding, black plastic containers modified with a light bulb fitted in the bottom portion of the container and a cage of mosquitoes placed in the top portion were used as experimental containers in this study (Additional file 1: Fig. S1). This study had two controls, one of which consisted of a container with no light bulb which served as a control for the light treatments (hereafter referred to as “light control”), showing what the baseline feeding is in the absence of a light treatment. The second control consisted of a cage placed on the bench outside of the containers with no light treatment, serving as a control for the temperature and humidity of the containers themselves (hereafter referred to as “container control”). The purpose of the two controls was to test if the containers themselves affect feeding outside of the light treatment. To test if containers have a consistent temperature, Thermochron I-buttons (Thermochron 2023) were deployed to measure temperature and relative humidity (RH).

**Fig. 1:**
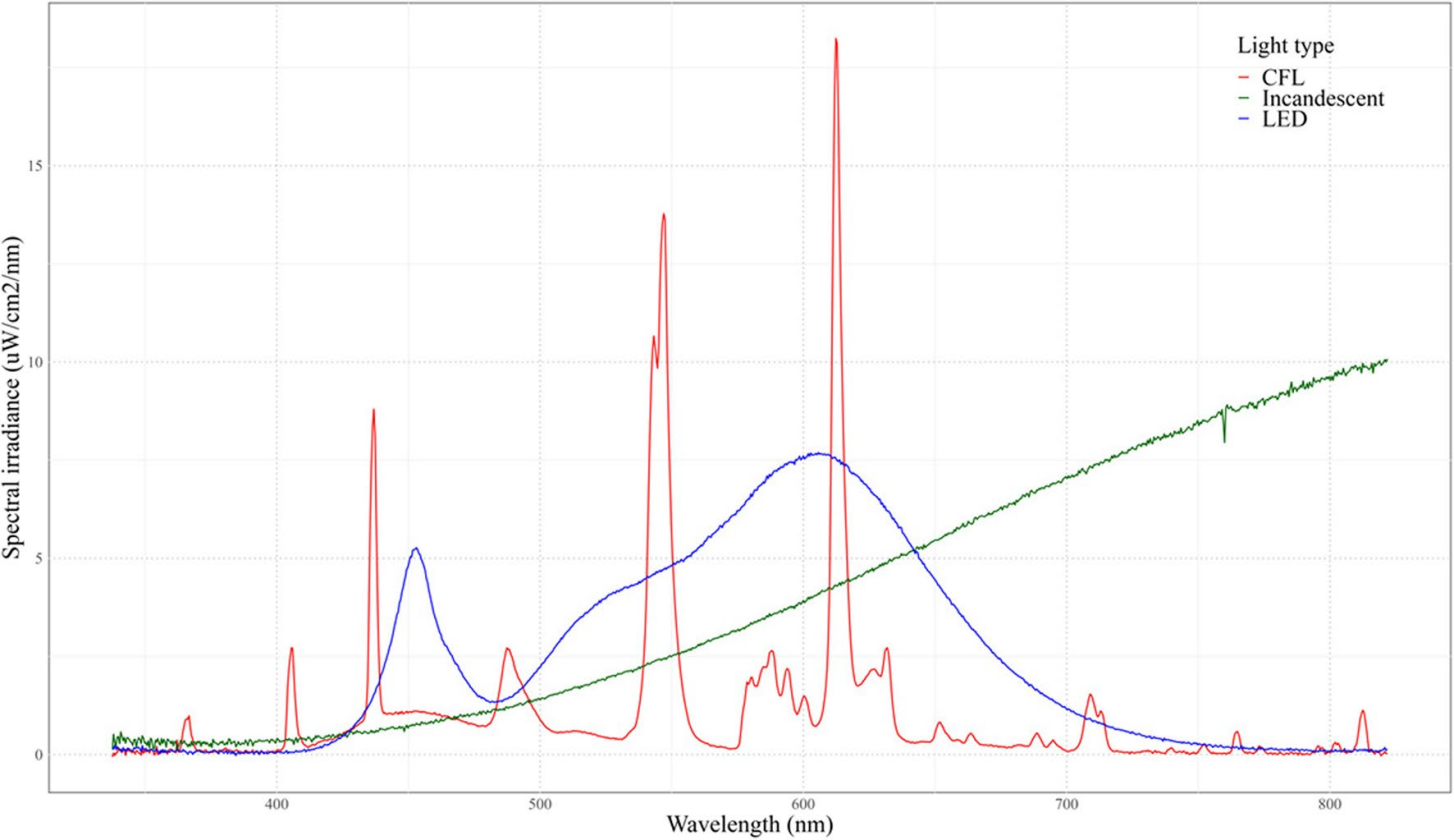
Emission profile showing the spectral irradiance (uW/cm.^2^/nm) versus wavelength (nm) of each of the three household lights used in this study (compact fluorescent light (CFL), light-emitting diode (LED), and incandescent light)

To quantify the characteristics of the lights used in this study, the spectra and intensity of three household lights (namely LEDs, CFLs, and incandescent lights) were measured. Each light was installed in an experimental container and spectral irradiance measurements were recorded using an Ocean Insight ST-VIS Miniature Spectrometer (Ocean Insight 2023a). Analysis of the lights was then performed using the OceanView software (Ocean Insight 2023b) and R (R Core Team 2022) to construct a spectral irradiance versus wavelength graph for each light (Fig. 1).

### Determining the effect of ALAN on *An. funestus* feeding

#### Biological material

*Anopheles funestus* colony (FUMOZ) originally established by colonizing a wild population from southern Mozambique (Hunt et al. 2005) maintained under standard laboratory conditions (Hunt et al. 2005) at the National Institute for Communicable Diseases facilities was used in this study. These facilities make use of a 12–12 light–dark cycle with two 30-min transition periods (“sunrise” and “sunset”) between the phases (Additional file 1: Fig. S2A). The colony is kept at 80% (± 10%) humidity and a temperature of 25 °C (± 2 °C). Larvae are fed a mixture of crushed dog biscuits and brewer’s yeast (Zengenene et al. 2022) and, prior to the isolation of the mosquitoes from the colonies for the experiments, 10% sucrose solution was supplied ad libitum to allow for the survival of adult mosquitoes.

#### Experimental design

*Anopheles funestus* mating is optimal from 10 days, with maximal mating success seen at 12 days (Maharaj et al. 2022). As unmated females would not be attracted to blood-feeding with higher feeding rates seen in older females, 11–12-day-old adult mosquitoes were used in this study to optimize the feeding rate (Maharaj et al. 2022; Aswat et al. 2023). A sample of 400–500, 11–12-day-old adult mosquitoes were isolated from the colonies for each biological replicate, placed into a large cage (W32.5 × D32.5 × H32.5 cm), and starved of sugar-water for 24 h before the start of the experiment. These individuals were then divided among ten small cages (W14 × D14 × H14 cm) each containing 40–50 individuals. We assumed a 50:50 sex ratio as no sex distortion has been reported (Zengenene et al. 2022) and, therefore, each cage contained 20–25 mated, unfed females.

The cages were exposed to the experimental treatments (Additional file 1: Fig. S2B). Zeitgeber time (ZT) was used, where ZT0 indicates the start of the “sunrise” transition period (i.e., the start of the subjective day) when lights start a brightening cycle, with ZT12 indicating the start of the “sunset” transition period (i.e., the start of the subjective night) when lights start a dimming cycle. The mosquitoes were allowed to undergo their normal “sunset” transition period from ZT12 to ZT12.30. Once complete darkness had set in at ZT12.30 and the mosquitoes had entered their night phase, the cages were placed into the experimental containers for 1 h of acclimation from ZT12.30 to ZT13.30. The experimental containers containing their individual treatments of either no light bulb (i.e., light control), LED bulb, CFL bulb, or incandescent bulb, as well as a cage placed on the bench outside the experimental containers (i.e., container control), were then exposed to their treatments for 30 min from ZT13.30 to ZT14.00. Each treatment had two technical replicates in every biological replicate. A total of six biological replicates were conducted, yielding a total sample size of 1656 female mosquitoes.

#### Blood feeding assay

Immediately after the light treatments, a standard artificial membrane feeding assay using a Hemotek membrane feeding system for blood-sucking insects (Hemotek® model PS6240, Hemotek Ltd, UK) was performed. Parafilm was used to cover the feeder plates, and defibrinated cattle blood from an accredited abattoir was added to the plates and the feeder plates were then connected to the heating unit set to 37 °C. The feeder plates were placed on top of the cages and feeding was allowed to take place for 30 min in complete darkness. After the completion of the blood-feeding assay, the number of dead females was recorded to assess mortality during the light treatments. The remaining live mosquitoes were aspirated from their cages into paper cups covered by netting and placed into a fridge set to 4 °C for 20 min to immobilize them. The number of “fed” and “unfed” females was then counted and recorded by examining their abdomen for the presence of blood (Rund et al. 2020; Aswat et al. 2023). The fed cohort included those that were fully fed as well as partially-fed.

#### Data analysis

To establish the effect of the experimental containers on mosquito mortality, an analysis of variance (ANOVA) was used to determine whether the mortality seen in the light control and the light treatments was significantly different from that seen in the container control. To establish whether there were significant differences in feeding between the biological replicates, an ANOVA followed by post hoc Tukey tests was performed. To determine whether the light treatment influenced the percentage of blood-fed females, another set of ANOVA and post hoc Tukey tests was conducted. For all three ANOVA tests, the assumptions of the tests were met, with the data being normally distributed (Shapiro–Wilk test, *p* > 0.05). To determine how the percentage of blood-fed females differs between treatments, the following formula was used to determine the percentage alteration of feeding: % alteration of blood-fed females = (% blood-fed females in light control) − (% blood-fed females in treatment). In order to establish what caused the effect on feeding status, a generalized linear model (GLM) was fitted. All statistical analyses were performed in R (R Core Team 2022).

## Results

### Assessing experimental conditions

Both temperature and RH fluctuated during the experimental period (Additional file 1: Fig. S3 and Fig. S4). The percentage of female mortality in the light control was 3.16% and that of the container control was 2.07% (Fig. S5). Using the Abbott formula to correct the mortality, the percentage of female mortality in the light control was 1.11%, incandescent 2.56%, CFL 2.13%, and LED − 0.62% relative to the container control. We tested for significant differences in the percentage of female mortality in the different treatments versus the two controls and could not detect any differences (ANOVA, *F* = 1.083, *p* = 0.386). Therefore, although an increase in temperature and a decrease in RH were seen during the experimental period, it was not significant; thus, it was concluded that the experimental containers had no direct effect on female mortality of the treatments versus the controls, and so heating was not sufficient to alter outcomes (Additional file 1: Fig. S5).

The emission spectrum of the CFL light contains several peaks across wavelengths from 300 to 700 nm while the LED light only shows two peaks, one at 450 nm and another from 500 to 700 nm (Fig. 1). The incandescent light shows a typical incandescent spectral signature, initially emitting 400-nm wavelengths that increase in wavelengths as the filament of the light gets hotter (Fig. 1). Therefore, the three household lights showed the typical spectral signatures and were classified as CFL, LED, and incandescent lights, respectively.

### Determining the effect of ALAN on *An. funestus* feeding

**Fig. 2:**
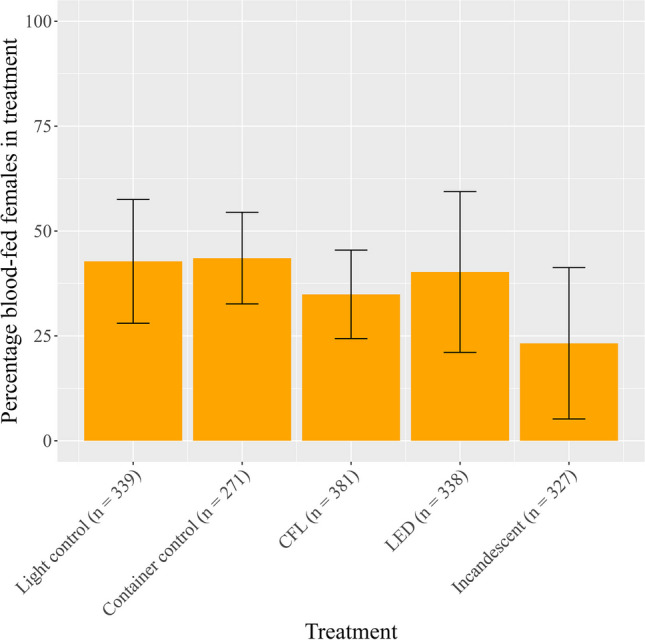
Bar graph of *Anopheles funestus* (Diptera: Culicidae) female mosquito feeding rate (%) at various light treatments ($\pm$ standard deviation, sd). Light treatments consisted of compact-fluorescent light (CFL), light-emitting diode (LED) light, and incandescent light. Controls included a container with no light (light control) and a cage fed outside of the container (container control). Sample sizes in each treatment are indicated in brackets next to treatment names

The percentage of blood-fed females across all biological replicates in the container control, light control, LED, CFL, and incandescent light treatments was 43.5%, 42.8%, 40.2%, 34.9%, and 23.2% respectively (Table 1). The percentage of blood-fed female *An. funestus* was reduced in all light treatments compared to the no-light control, with the largest reduction of 19.6% seen under the incandescent light treatment (Table 1, Fig. 2). The percentage of females that fed in the container control (43.5%, standard deviation (sd): 10.9%) and light control (42.8%, sd: 14.8%) was similar, indicating that the experimental containers had no significant effect on mosquito blood-feeding (Table 1). Using ANOVA and post hoc Tukey tests, no statistically significant differences in the feeding rate of female mosquitoes exposed to the different light treatments could be detected (ANOVA, *F* = 1.948, *p* > 0.05) (Additional file 1: Table S1, Table S2). Only the container control shows a significant difference in the percentage of partially and fully blood-fed females (ANOVA, *F* = 6.547, *p* = 0.028) (Additional file 1: Fig. S6, Table S3).

**Table 1:** Proportion (%) of blood-fed female *Anopheles funestus* mosquitoes in the various treatments, with standard deviation (sd) and sample sizes for each treatment and controls

Treatment	Fed (%)	sd	Sample size
Light control	42.8	14.8	339
LED light	40.2	19.2	338
CFL light	34.9	10.5	381
Incandescent light	23.2	18.0	327
Container control	43.5	10.9	271

Three GLMs with either treatment, biological replicate, or both treatment and biological replicate as predictor variables of feeding status as the response variable were fitted (Additional file 1: Table S4). The GLM containing only treatment as the predictor of feeding status revealed treatment as a significant predictor (ANOVA, *p* < 0.05), with the incandescent light treatment as the only significant predictor of feeding status (GLM, estimate =  − 0.407, *p* < 0.05) (Table 2 and Additional file 1: Table S5). The GLM containing both treatment and feeding status revealed only treatment as the significant predictor (ANOVA, treatment *p* < 0.05, biological replicate *p* = 0.45). The GLM containing only biological replicate (Additional file 1: Table S6) was not significant (ANOVA, *p* = 0.55). A biological replicate was explored as a predictor variable to confirm that there was no significant difference in the percentage of blood-fed females in a given treatment across biological replicates. The lowest Akaike information criterion (AIC) score was seen in the model containing treatment as the only predictor variable of feeding status (Additional file 1: Table S4). This model only explained 0.1% less variation in the data (Additional file 1: Table S4) than the model containing both treatment and biological replicate as predictors. Therefore, the additional variable does not explain more variation and so only treatment as a predictor variable was retained.

**Table 2:** Estimated regression parameters, standard errors, *z*-values, and *p*-values for the Poisson GLM containing treatment presented in Table S4

	Estimate	Standard error	*z*-value	*p*-value
Intercept	− 1.052	0.087	− 12.138	** < 2e-16**
Treatment light control^a^	0.203	0.120	1.692	0.091
Treatment container control^a^	0.221	0.126	1.748	0.081
Treatment incandescent^b^	− 0.407	0.144	− 2.829	**0.005**
Treatment LED^a^	0.142	0.122	1.165	0.244

^*^Superscripts are used to indicate significant differences; bold entries indicate significant *p*-values i.e., *p* < 0.05

## Discussion

To our knowledge, this study is the first to demonstrate an overall reduction in *An. funestus* feeding following light exposure. However, unlike previous work on other species (Burkett and Butler 2005; Rund et al. 2020), the effect sizes are relatively smaller. Sheppard et al. (2017) demonstrated that a 10-min pulse of 300 lx LED white light significantly reduces the night-time feeding of nocturnal *An. gambiae* by 42%. Rund et al. (2020) showed that a 30-min pulse of 50 lx incandescent white ALAN significantly increases the percentage of night-time feeding of the diurnal *Aedes aegypti* mosquito. Rund et al. (2020) illustrated that the group exposed to ALAN had a twofold increase in feeding during the night compared to the group maintained in their normal light–dark cycle. The experiments in this study were based off the findings by Rund et al. (2020) and Sheppard et al. (2017), where the authors exposed mosquitoes to a 30-min and 10-min light pulse respectively, with a human arm feeding assay run immediately following the light treatment. Although the current study and that by Rund et al. (2020) and Sheppard et al. (2017) have important methodological differences such as the length of the light stimulus and blood source and delivery, the experimental outline is roughly the same where all three studies test the immediate inhibition of mosquito blood-feeding by a light stimulus. Therefore, a significant decrease in the percentage of blood-fed nocturnal *An. funestus* females was expected, similar to the decrease in feeding seen in the nocturnal *An. gambiae* (Sheppard et al. 2017) and the increase in night-time feeding seen in the diurnal *Aedes aegypti* (Rund et al. 2020). The results of the current study, however, differ from that of previous work.

The similarity in feeding rate between treatments in this study could be due to several reasons. As *An. funestus* has been found to show a lower feeding rate on artificial membranes compared to live hosts (Niain’ny Felamboahangy et al. 2023), the use of artificial membrane feeding systems in this study, as opposed to human arm feeding assays in previous work, could contribute to the observed variations due to methodological differences in blood source and delivery. The large biological variation in feeding observed in this study aligns with findings that membrane-feeding systems show larger standard deviations around mean feeding percentages compared to live hosts (Niain’ny Felamboahangy et al. 2023). The wide biological variation in feeding success could also indicate that the colony is not consistently feeding well on the artificial membrane system, a well-known challenge when working on *An. funestus*. Differences in the intensity and spectra of the household lights used here and that of the incandescent light used by Rund et al. (2020) and the LED light used by Sheppard et al. (2017) could also account for the variations seen. As different species, and in one case genus, were investigated by previous work and the current study, biological/behavioral differences related to feeding may account for the variations observed (Sherrard-Smith et al. 2019; Baik et al. 2020). Even within the same species, *An. funestus* strains from different geographic regions show large variations in reproductive success and genomic differences which have been shown to affect fitness, for example (Ngowo et al. 2021; Odero et al. 2023; Mrosso 2024). Therefore, variations seen in this study versus previous work may be due to species, genetic, and geographic variations.

The finding that only incandescent light significantly suppresses feeding suggests that commonly used household lights, at lower intensities and more “real-world” spectra, may not have the same impact on mosquito behavior as seen under laboratory conditions in other mosquito species. LEDs are becoming more widespread due to their affordability and energy efficiency compared to incandescent lights (Wilson et al. 2021). The higher electricity consumption of energy-inefficient incandescent lights results in increased economic costs. Thus, given the prevalence of malaria in low-income regions, the economic burden of using inefficient lights may outweigh the potential public health benefits of reducing malaria. Therefore, the costs and benefits of implementing such a strategy should be evaluated carefully, with regional considerations playing a critical role.

The light pulse in this study occurred from 19h30 (ZT13.30), a time when people are typically awake and using ALAN but are not yet protected by bed nets. As *An. funestus* feed optimally from 22h00 (ZT16), future studies could explore the impact of light pulses at 22h00 and later. This would help determine if the time of light exposure significantly influences the feeding behavior of *An. funestus*. As colonized *An. funestus* mosquitoes were used in this study, future work should evaluate the effect of ALAN on wild mosquitoes as they might display different preferences than laboratory-reared mosquitoes. The effect of ALAN on mosquito feeding should also be assessed under field or semi-field conditions as the environment, and the associated structures and vegetation, will affect the light conditions that the mosquitoes are exposed to via reflection, refraction, and absorption of light. Nonetheless, the change in innate mosquito feeding behavior by ALAN may result in population- and, ultimately, community-level changes in ecosystems (Coetzee et al. 2023).

## Conclusion

Our results suggest that exposure to some artificial lights found in households during the night may have an immediate inhibitory effect on *An. funestus* feeding. By helping identify which light types lead to a suppression of feeding, the findings of this study could provide insight necessary to design household lights that help minimize mosquito feeding on humans. Further research into the spectral sensitivities of *Anopheles* species and the effect of light of different intensities and spectra on feeding behavior across mosquito species, in real-world systems, is essential before light can be implemented as a complementary malaria transmission reduction strategy.

## Supplementary information

Below is the link to the electronic supplementary material.

Supplementary file1 (DOCX 1312 KB)

## Data Availability

The data generated from experiments in this study are available as Additional file 2. Raw data is available upon request.

## Abbreviations

AIC: Akaike information criterion
ALAN: Artificial light at night
*An*.: *Anopheles*
ANOVA: Analysis of variance
CFLs: Compact fluorescent lights
LEDs: Light-emitting diodes
RH: Relative humidity
sd: Standard deviation
s.s.: Sensu stricto
ZT: Zeitgeber time
