# Supplementary material for: Only incandescent light significantly decreases feeding of Anopheles funestus s.s. (Diptera: Culicidae) mosquitoes under laboratory conditions
Source: Parasitol Res. 2024 Oct 18;123(10):355. doi: 10.1007/s00436-024-08370-3 (PMC11489244; doi:10.1007/s00436-024-08370-3)
Supplement: Supplementary file 1 — Supplementary file1 (DOCX 1312 KB) [file 436_2024_8370_MOESM1_ESM.docx]

# Supplementary information: Additional file 1

| 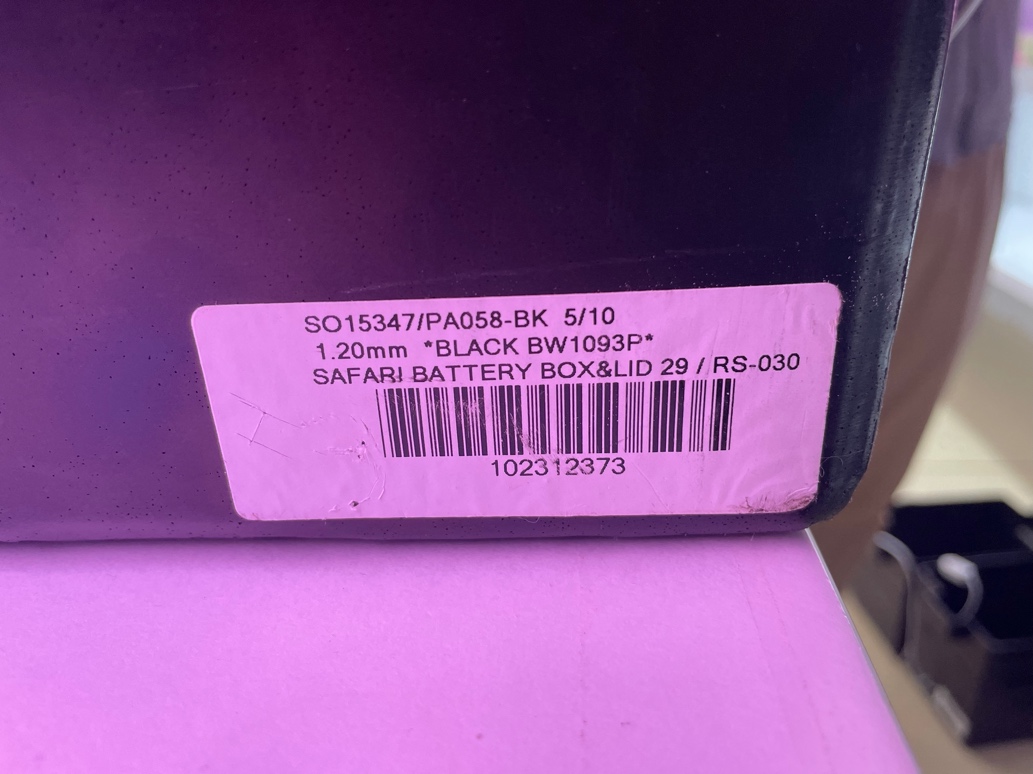 | |
| --- | --- |
| 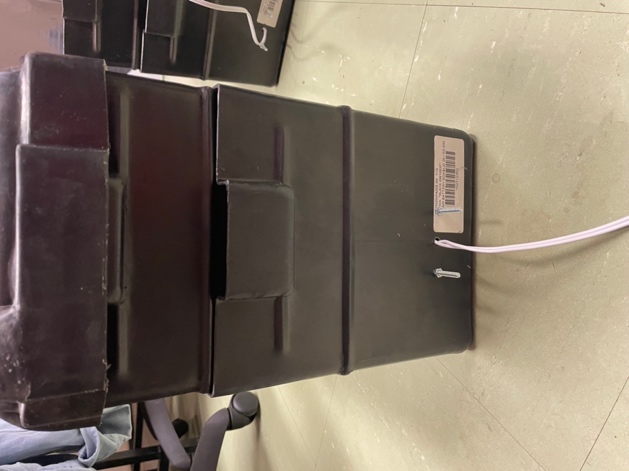 | 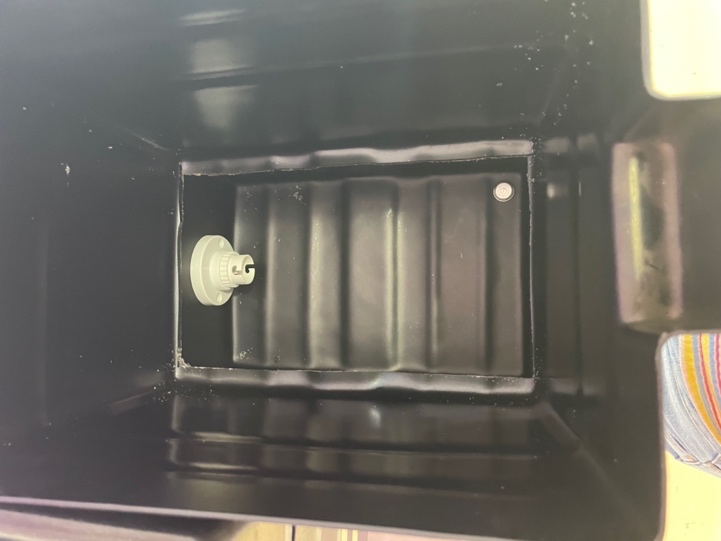 |
| 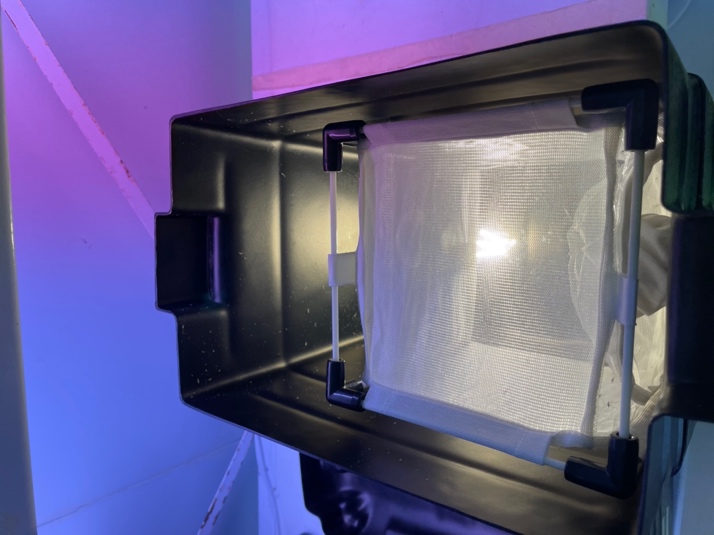 | 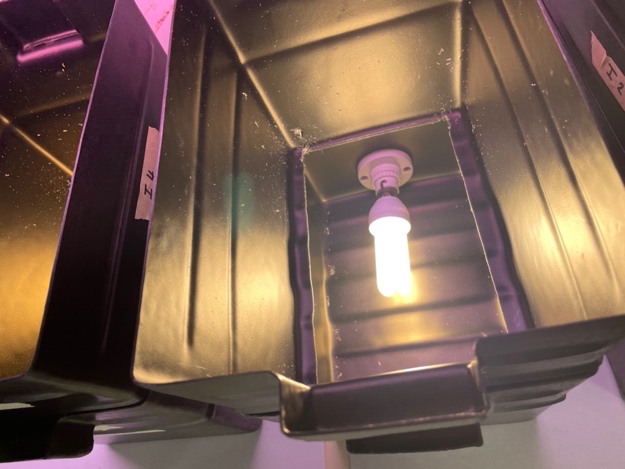 |

**Figure S1.** Images showing the design of the experimental containers.

Zeitgeber time (ZT, h)

‘sunrise’

‘sunset’

0

24

12

**Light-dark cycle maintenance conditions**

1. **MAINTENANCE CONDITIONS**


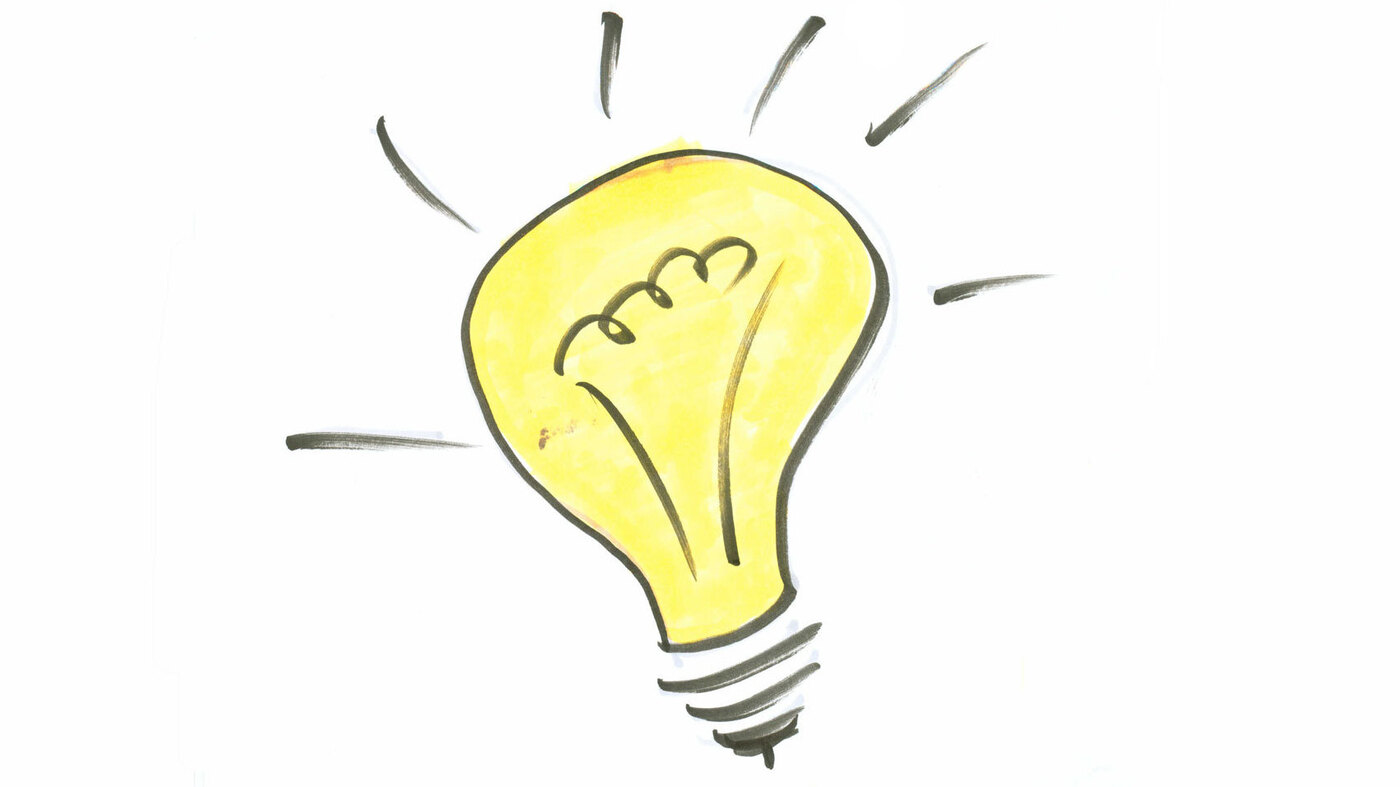


30-minute Feeding assay

ZT14.00

Light exposure ZT13.30

(30 minute)

1 hour acclimation

ZT12.30 – 13.30

**b. EXPERIMENTAL DESIGN**

Sunset

ZT12 – 12.30

**Light treatment**

30-minute Feeding assay

ZT14.00

1 hour acclimation

ZT12.30 – 13.30

Sunset

ZT12 – ZT 12.30

**Light control and container control**

**Figure S2.** The percentage of female *Anopheles funestus* feeding was determined by a standard membrane feeding assay using a Hemotek blood feeding system. (a) Standard maintenance conditions of *An.* *funestus* colonies. Bar represents the 12-12 light-dark cycle. ZT0 = start of ‘sunrise’, ZT12 = start of ‘sunset’. (b) Experimental design for the testing of the effect of different light treatments on the percentage of *An. funestus* feeding.

**
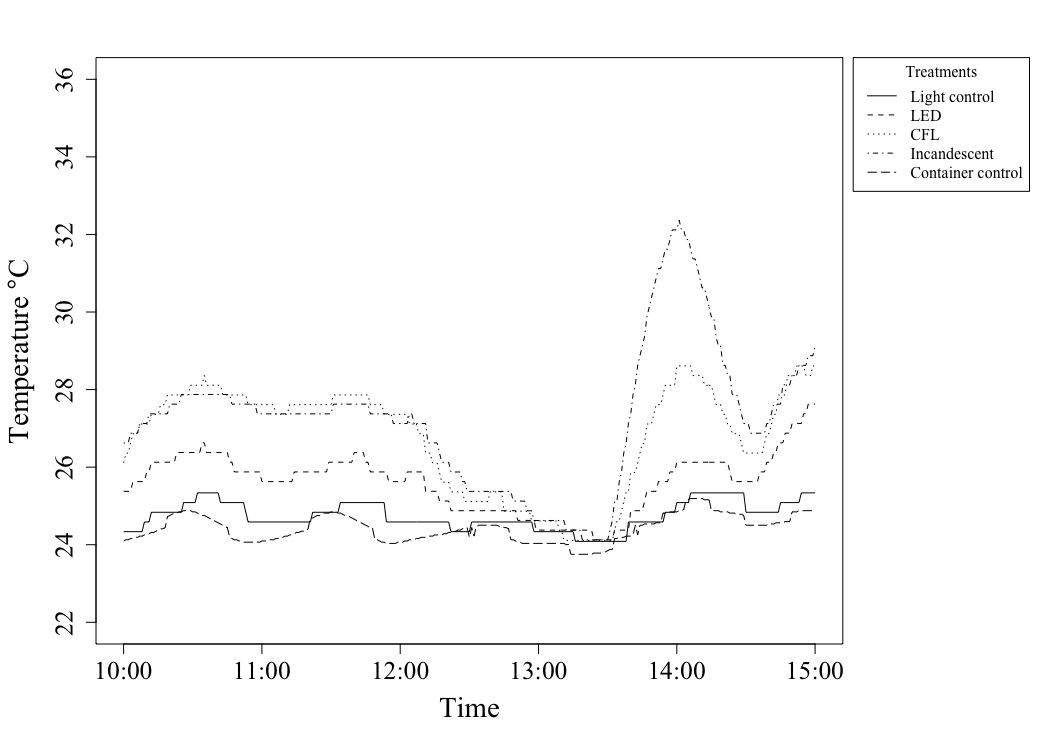
** **Figure S3.** Temperature (℃) in the experimental containers recorded with Thermochron I-buttons. The lighting conditions as well as the time of the light treatment and feeding assay, indicated by an arrow, are shown above the graph.


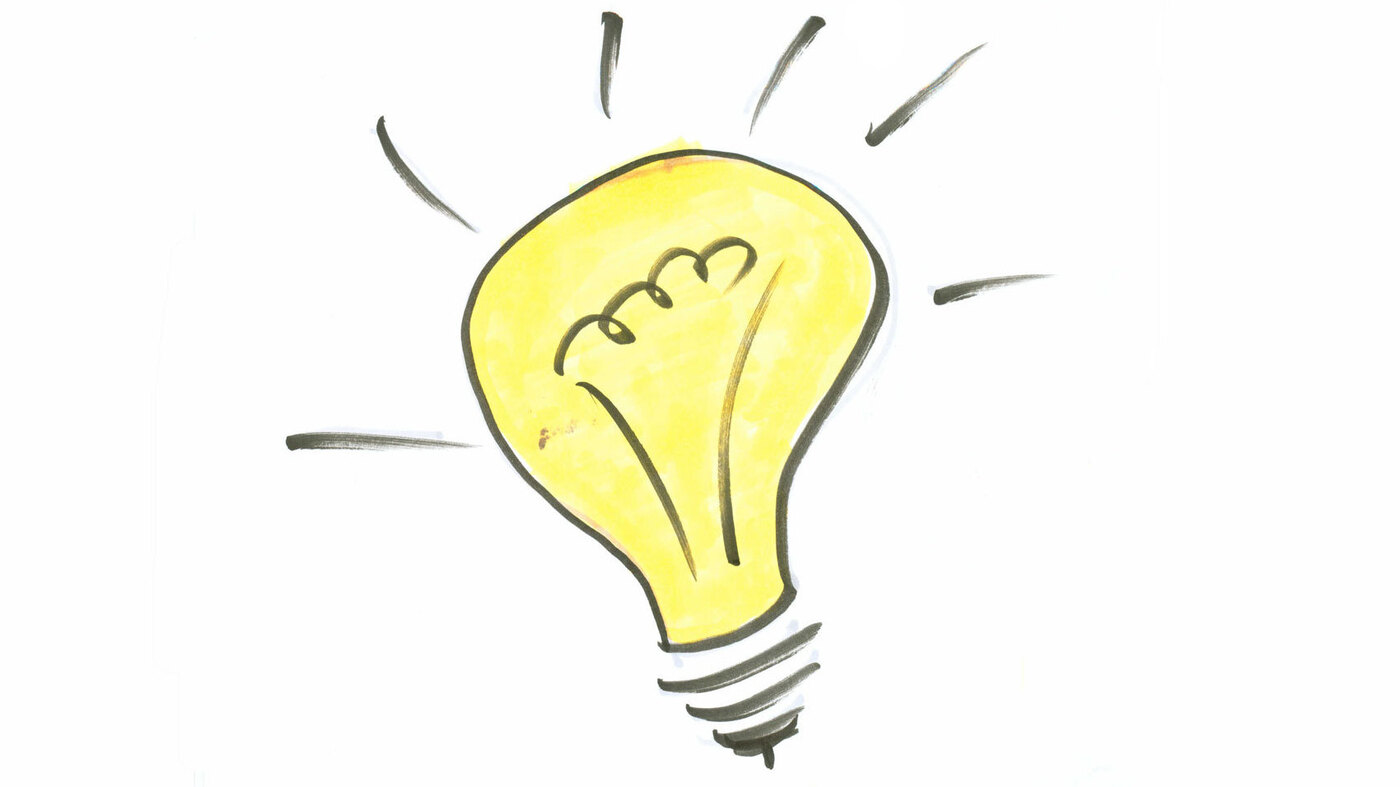


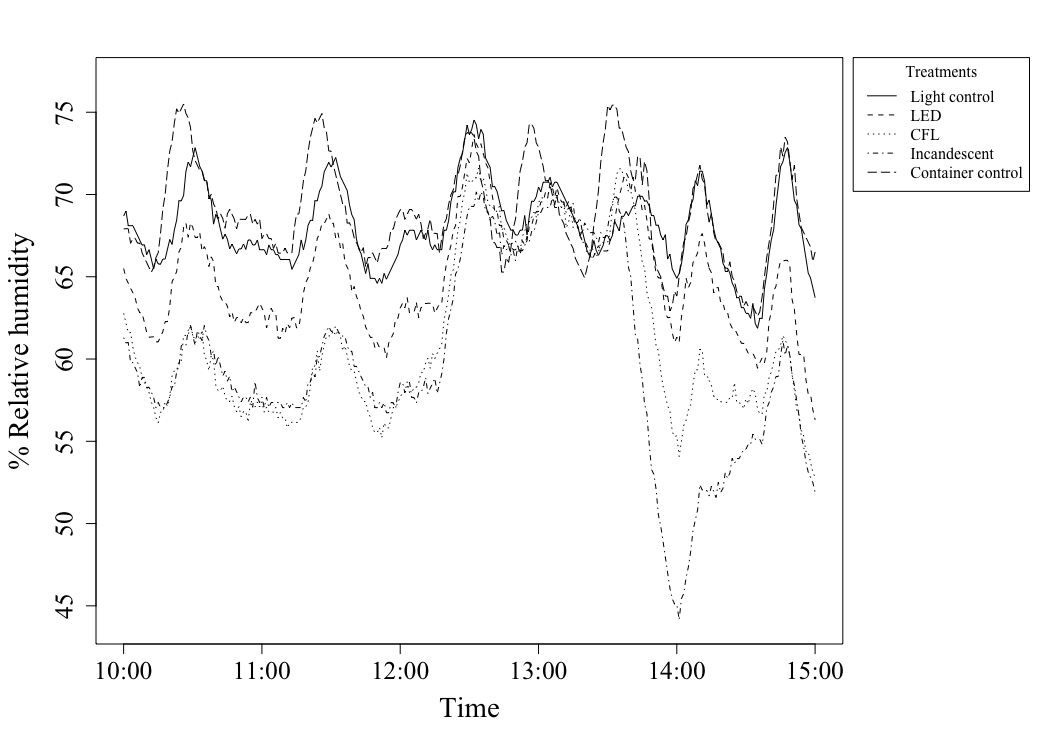


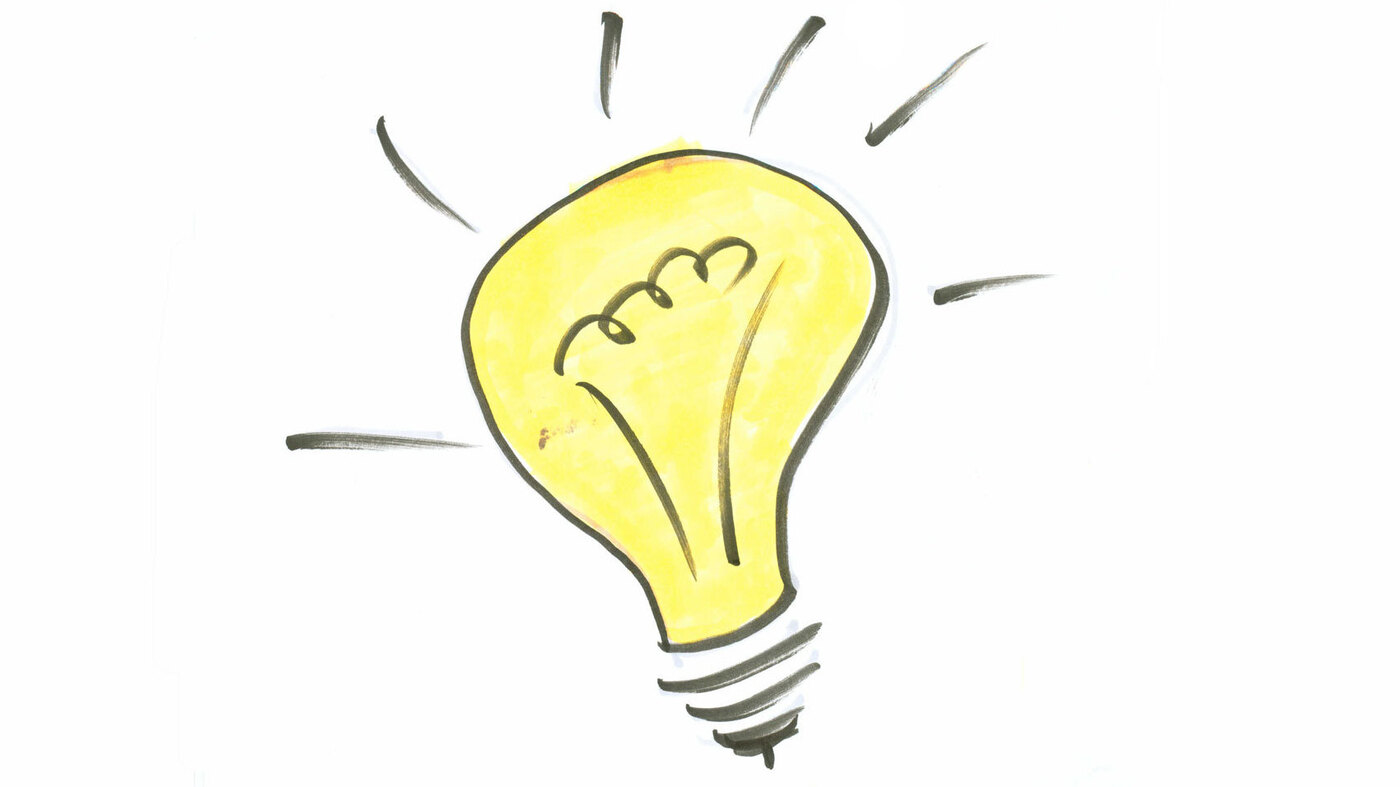


**Figure S4**. Relative humidity (%) in the experimental containers recorded with Thermochron I-buttons. The lighting conditions as well as the time of the light treatment and feeding assay, indicated by an arrow, are shown above the graph.


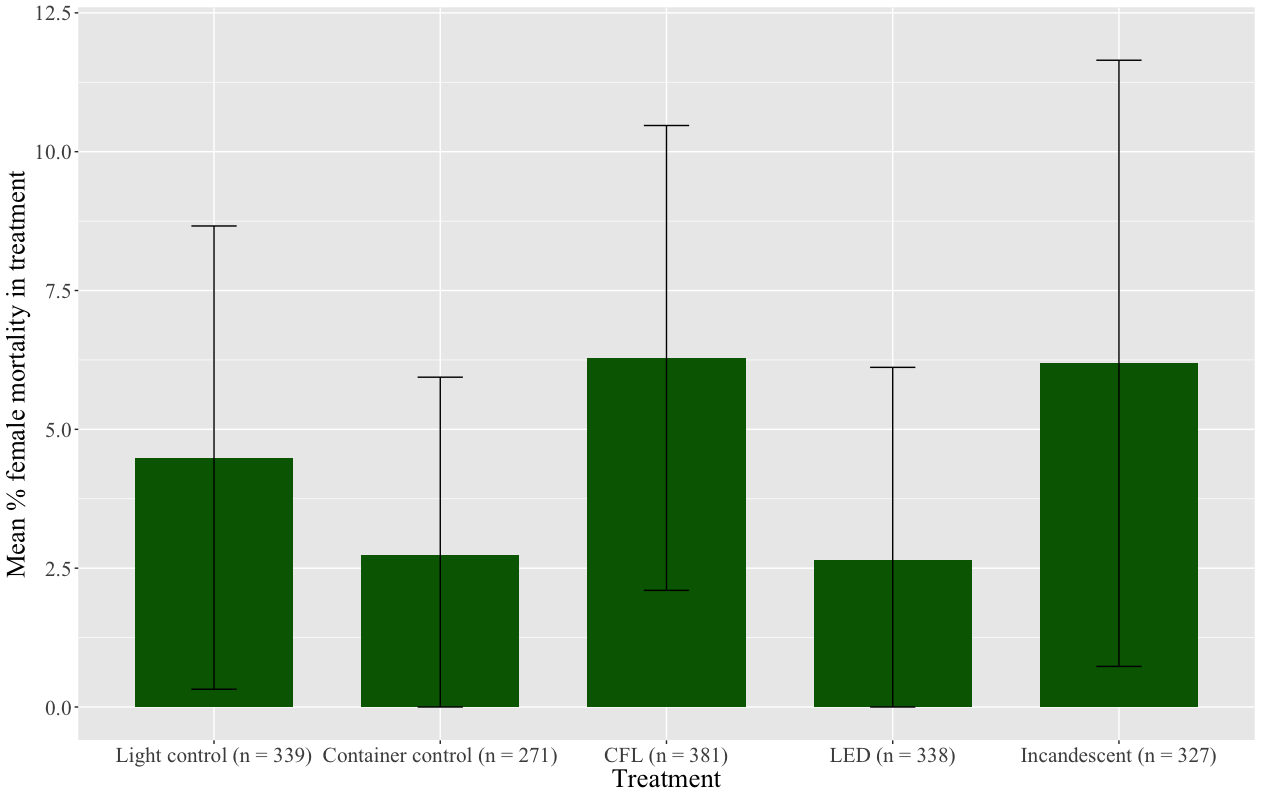


**Figure S5.** Bar chart of the average female mortality (%) in each treatment with sample sizes indicated in brackets next to the treatment names.


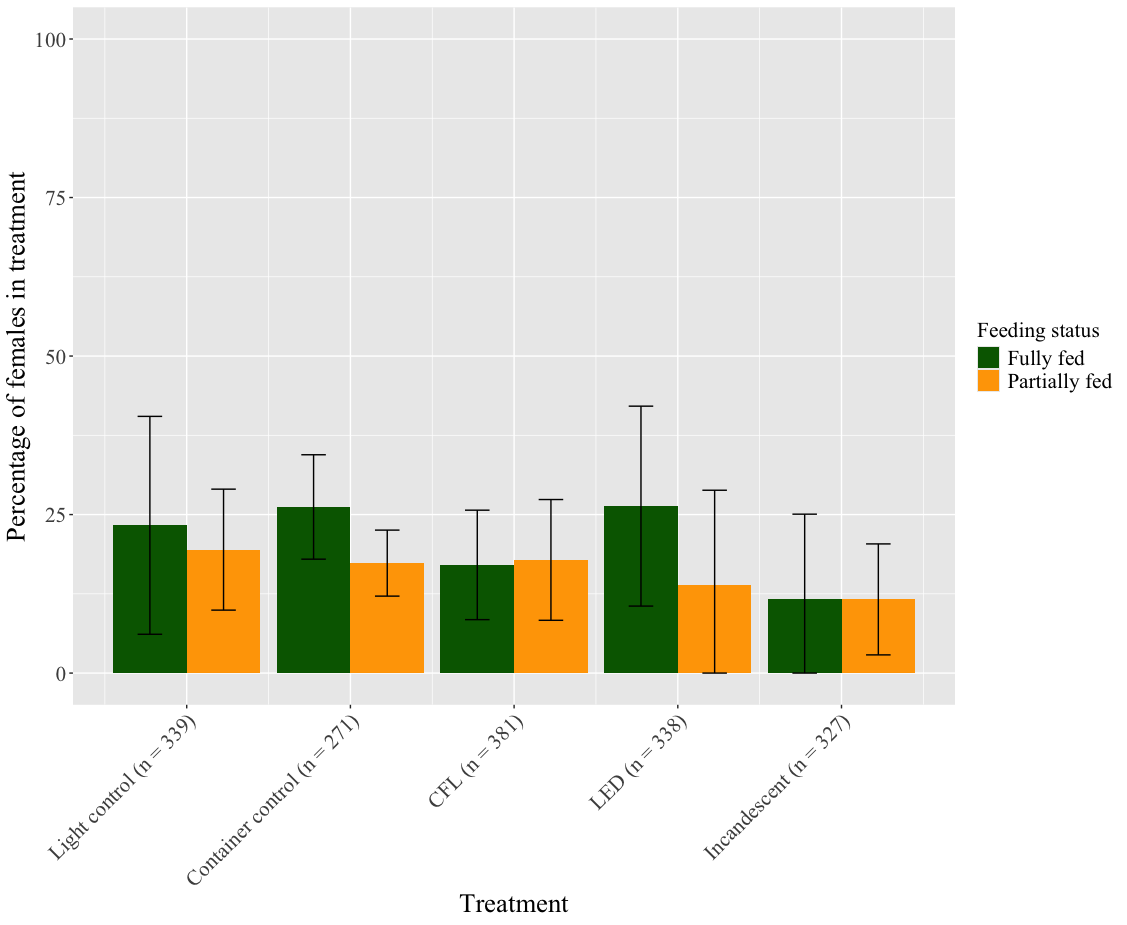


**Figure S6.** Bar graph of *Anopheles funestus* (Diptera: Culicidae) female mosquito feeding rate (%) at various light treatments ($\pm$ standard deviation, sd) showing partially as well as fully fed percentages. Light treatments consisted of compact-fluorescent light (CFL), light-emitting diode (LED) light and incandescent light. Controls included a container with no light (light-control) and a cage fed outside of the container (container-control). Sample sizes in each treatment are indicated in brackets next to treatment name.

**Table S1**. ANOVA results of the comparison of the percentage of blood-fed females in the various treatments.

|  | **Df** | **Sum Sq** | **Mean sq** | **F value** | **P (>F)** |
| --- | --- | --- | --- | --- | --- |
| Treatment | 4 | 1777 | 444.3 | 1.948 | 0.134 |
| Residuals | 25 | 5704 | 228.1 |  |  |

**Table S2.** Results of a *post hoc* Tukey test of the percentage of blood-fed females in the different treatments across all the biological replicates.

| **Comparison** | **Difference** | **Lower CI** | **Upper CI** | **P-Adjusted** |
| --- | --- | --- | --- | --- |
| Container control-CFL | 9.0870196 | -16.524395 | 34.698434 | 0.8334504 |
| Incandescent-CFL | -12.1418334 | -37.753248 | 13.469581 | 0.6379963 |
| LED-CFL | 2.8326443 | -22.778770 | 28.444059 | 0.9974264 |
| Light control-CFL | 8.4751338 | -17.136281 | 34.086548 | 0.8651980 |
| Incandescent-Container control | -21.2288530 | -46.840267 | 4.382561 | 0.1391089 |
| LED-Container control | -6.2543752 | -31.865790 | 19.357039 | 0.9505580 |
| Light control-Container control | -0.6118858 | -26.223300 | 24.999529 | 0.9999941 |
| LED-Incandescent | 14.9744778 | -10.636937 | 40.585892 | 0.4421043 |
| Light control-Incandescent | 20.6169672 | -4.994447 | 46.228382 | 0.1585556 |
| Light control-LED | 5.6424894 | -19.968925 | 31.253904 | 0.9656083 |

**Table S3.** ANOVA results of the comparison of the percentage of partially and fully blood-fed females in the various treatments.

| **Treatment** | **F value** | **p-value** |
| --- | --- | --- |
| CFL | 0.08467537 | 0.77700469 |
| Container control | 6.54730885 | **0.02843304** |
| Incandescent | 0.02974643 | 0.86650750 |
| LED | 0.84795840 | 0.37880972 |
| Light control | 0.20761499 | 0.65837713 |

**Table S4**. Generalized linear models fitted with their AIC values, null deviance, residual deviance and estimated deviance explained by the models.

| **Model** | **AIC** | **Null deviance** | **Residual deviance** | **Deviance explained (%)** |
| --- | --- | --- | --- | --- |
| glm(`Feeding status` ~ Treatment, family = "poisson") | 2417.9 | 1218.4 | 1191.9 | 2.177 |
| glm(`Feeding status` ~ `Biological replicate`, family = "poisson") | 2438.1 | 1218.4 | 1218.1 | 0.030 |
| glm(`Feeding status` ~ Treatment + `Biological replicate`, family = "poisson") | 2419.3 | 1218.4 | 1191.3 | 2.224 |

**Table S5.** Estimated regression parameters, standard errors, z-values and P-values for the Poisson GLM containing treatment and biological replicate presented in Table S3.

|  | **Estimate** | **Standard error** | **z-value** | **p-value** |
| --- | --- | --- | --- | --- |
| Intercept | -1.124 | 0.129 | -8.724 | **< 2e-1** |
| Treatment Light control | 0.206 | 0.120 | 1.716 | 0.086 |
| Treatment Container control | 0.227 | 0.127 | 1.790 | 0.074 |
| Treatment Incandescent | -0.407 | 0.144 | -2.827 | **0.005** |
| Treatment LED | 0.138 | 0.122 | 1.134 | 0.257 |
| Biological replicate | 0.019 | 0.025 | 0.752 | 0.452 |

**Table S6**. Estimated regression parameters, standard errors, z-values and P-values for the Poisson GLM containing biological replicate presented in Table S3.

|  | **Estimate** | **Standard error** | **z-value** | **p-value** |
| --- | --- | --- | --- | --- |
| Intercept | -1.057 | 0.101 | -10.422 | **<2e-16** |
| Biological replicate | 0.015 | 0.025 | 0.599 | 0.549 |

# Additional file 2:

**Dataset S1**. *Anopheles funestus* feeding rate (%) and female mortality (%) in each treatment.

| **Treatment** | **Number of fed females** | **Number of unfed females** | **Female mortality** | **Total female mosquitoes** | **% fed females** | **% female mortality** |
| --- | --- | --- | --- | --- | --- | --- |
| Light control | 145 | 194 | 15 | 339 | 42,77286136 | 4,171149 |
| LED | 136 | 202 | 7 | 338 | 40,23668639 | 3,468018 |
| CFL | 133 | 248 | 22 | 381 | 34,90813648 | 4,185582 |
| Incandescent | 76 | 251 | 22 | 327 | 23,24159021 | 5,457893 |
| Container control | 118 | 153 | 9 | 271 | 43,54243542 | 3,195751 |
